# Supplementary material for: Influence of open-source virtual-reality based gaze training on navigation performance in Retinitis pigmentosa patients in a crossover randomized controlled trial
Source: PLoS One. 2024 Feb 1;19(2):e0291902. doi: 10.1371/journal.pone.0291902 (PMC10833541; doi:10.1371/journal.pone.0291902)
Supplement: S1 Appendix — (PDF) [file pone.0291902.s004.pdf]

Appendices

Appendix A - Gaze pattern evaluation and Multimatch-algorithm

As was described in the 'Suggested gaze pattern' section, the patients' gaze movements during training were measured, and the similarity between this gaze movement and the suggested gaze pattern was calculated at run-time. To do so, the first step is to analyze the eye-tracking data captured by the VR device to determine saccades, as described in the section 'Saccade characteristics and gaze pattern similarity'. Using a modified Multimatch-Algorithm [32] , sections of multiple saccades executed by the participant are compared to a saccade representation of the suggested gaze pattern (Fig. 12) to calculate a similarity value based on how well the two saccade patterns match. This similarity value was displayed to participants after each trial of the Gaze Training, giving them a quantitative measure of how closely their gaze movements match the suggested gaze pattern.

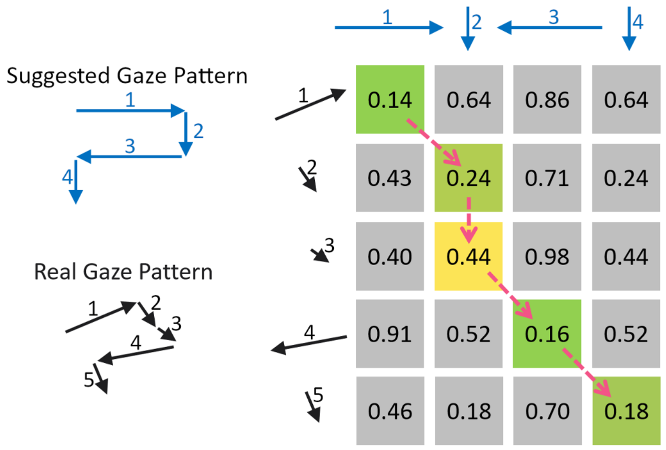

Figure 12: Visualization of a Multimatch-based comparison between a gaze pattern displayed by a participant (Real Gaze Pattern) and an ideal representation of the suggested gaze pattern. Each square displays the difference in angle and amplitude between the respective saccade vectors, with 0.0 meaning saccades are identical and 1.0 meaning saccades are complete opposites. Colorized squares indicate the "path of the least resistance" determined by the Multimatch-algorithm, which describes the best match between the two patterns.

Appendix B - Patients’ visual fields

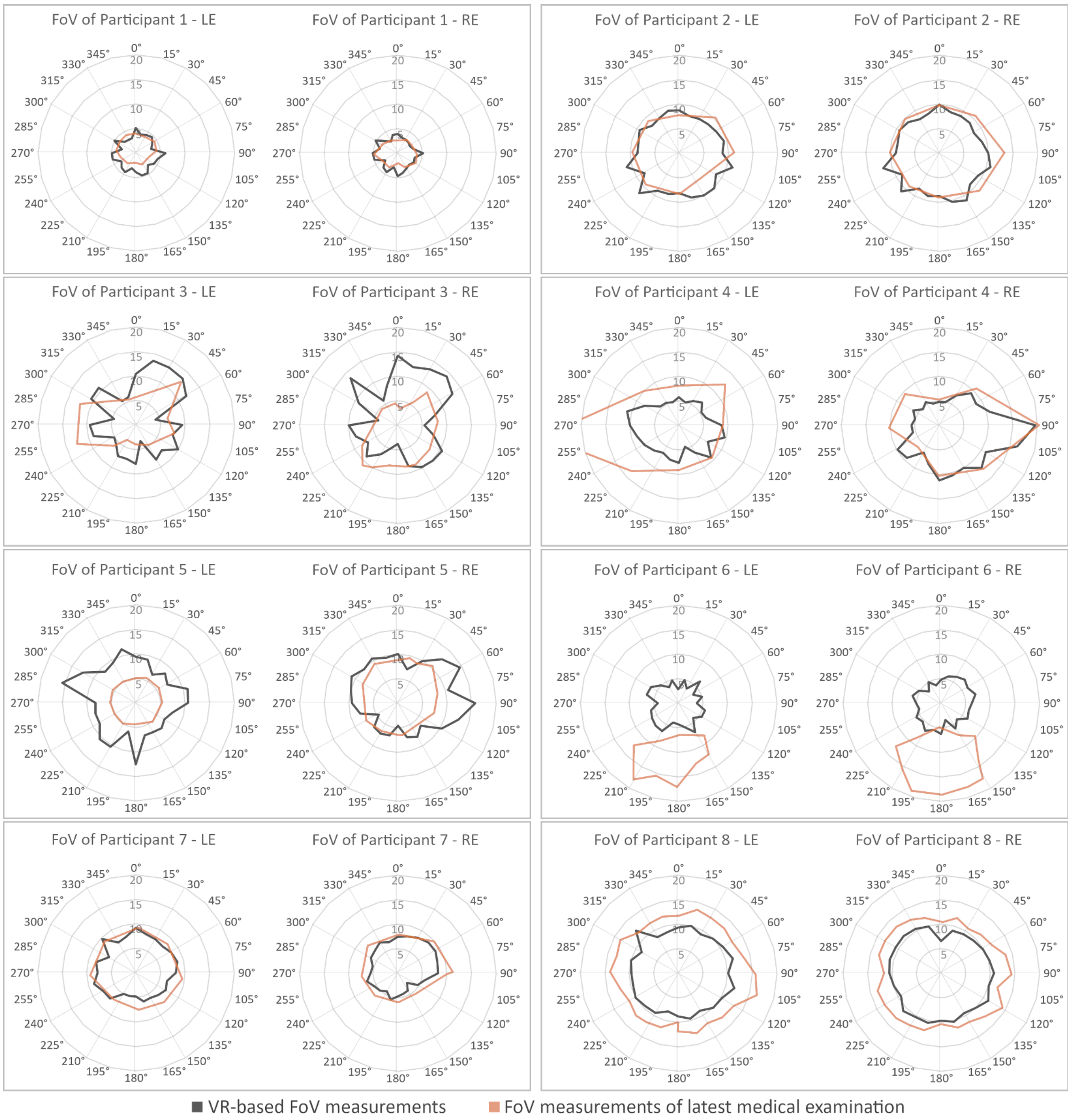

Figure 13: Visualization of the visual field dimensions of the eight participants who completed the study. Grey indicates the VF measured by the self-developed, VR-based kinetic perimetry tool as described in section 3.2.2, orange indicates the participants’ VF based on their most recent medical examination.

Appendix C - Dynamic visual field calculation, saccade detection, and saccade characteristics

Calculaton of the dynamic visual field The dynamic visual field (DVF) served as important measurement parameter to explore the visual performance of participants in this study. As outlined in the section ’Real-world obstacle course measurements’, the DVF describes the visual area that a person with VFD observes within a specific time interval utilizing their head- and eye movements. This section will provide a detailed explanation of the process for calculating and computing the DVF.

Initially in the computational analysis of the DVF, a virtual spherical grid is defined around the user, with the eye tracker in its center. The grid is subdivided into sections of

1° horizontal and vertical angle, resulting a two-dimensional array consisting of $360 * 180$ individual sections. Next, the eye tacking data, which was captured both within the VR training as well as in real-world trials, is analyzed. For the calculation of the DVF, three parameters are extracted from each eye tracking sample: The time stamp at which the sample was captured, the elevation angle and azimuth angle of the gaze direction measured at that specific time. In addition to these parameters, the static VF size of the respective participant, as reported in table 1, is required for the calculation of the DVF. For each eye tracking sample, the current gaze direction is projected onto a point (x, y) within the two-dimensional array. If the participant's gaze is directed forward, the gaze direction would be mapped exactly in the center of the array at position $(180, 90)$. If the gaze then shifts, for example, by 10° to the right or left, the projected position on the array would change to $(190, 90)$ or $(170, 90)$, respectively. If the gaze shifts upwards or downwards by 10°, the resulting projected position in the array would be $(180, 100)$ or $(180, 80)$. The next step is to project not just the gaze direction to the grid, but the entire VF of the participant. In other words, it must be determined which sections in the array are currently covered by the participant's VF. To achieve this, the following formula is utilized: $\sqrt{(x_{grid} - x_{gaze})^2 + (y_{grid} - y_{gaze})^2} \leq r_{VF}$. Here, x_{grid} and y_{grid} describe the horizontal and vertical position of the section in the grid, x_{gaze} and y_{gaze} describe the projected position of the gaze in the grid, and r_{VF} is the average VF radius of the participant. Each section that is identified to be within the participants VF is annotated with the time stamp of the current eye tracking sample. If the section already contains a time stamp, the old time stamp is overwritten with the newer one. This results in each section of the array containing information about the last time stamp at which it was covered by the VF - or, in other words, the last time it was observed by the participant. The subsequent step to determine the DVF involves iterating through each individual section in the two-dimensional array, counting the number $n_{observed}$ of sections annotated with a time stamp that falls within the specified time interval. For example, with a specified interval of three seconds, $n_{observed}$ would include all sections with time stamps less than three seconds old. To enhance the interpretability of the DVF output, it is reported as a percentage of the visual area that could be observed by a static healthy VF with approximated dimensions of $180 * 135$. In summary, the DVF is calculated as $DVF = \frac{n_{observed}}{180*135} * 100\%$. This calculation is performed for every eye tracking sample measured within a trial. The average of all calculated values yields the DVF for the respective trial, reported in the supplementary file S1.

Saccade detection Fig. 14 visualizes the saccade detection approach described in the section 'Real-world obstacle course measurements' in the point 'Saccade characteristics and gaze pattern similarity'.

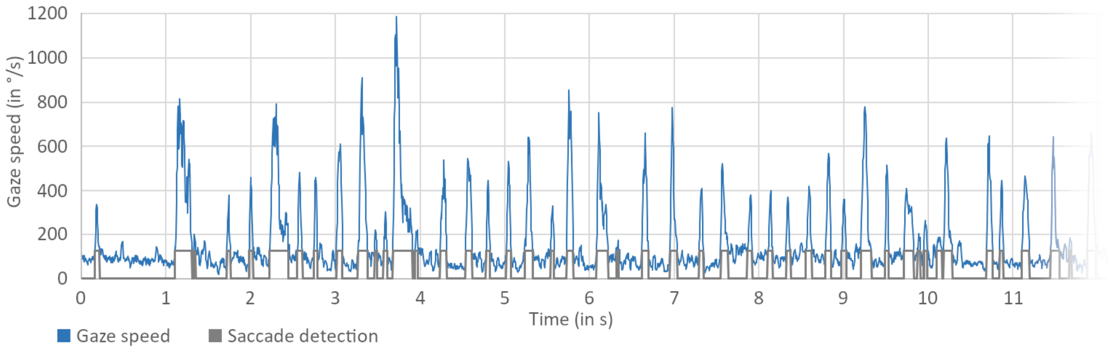

Figure 14: Visualization of the saccade detection following the algorithm of Nyström et al. [40]. The displayed data is taken from one of the real-world obstacle course trials, measured with the Pupil Labs Invisible eye tracker [36].

Saccade characteristics The results for the different saccade characteristics described in this section are shown in Fig. 15.

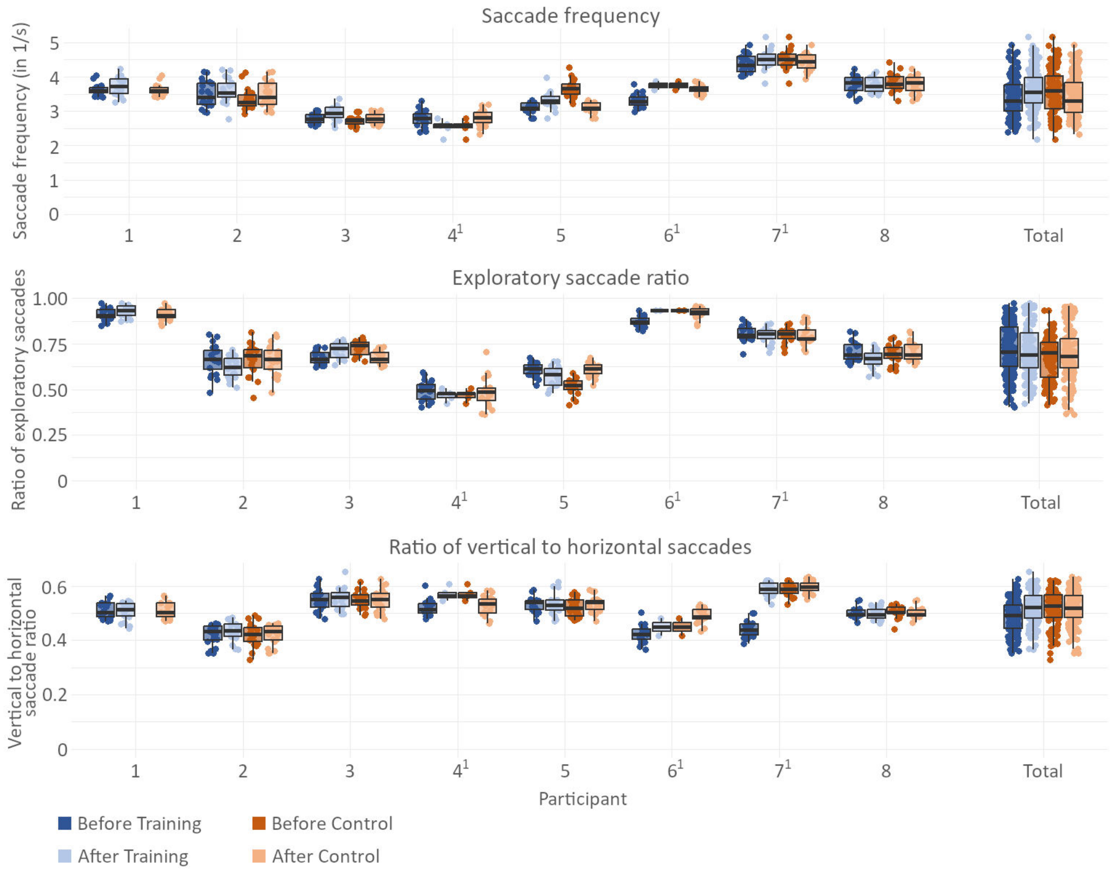

Figure 15: Results of the three parameters saccade frequency, ratio of exploratory saccades, and ratio between vertical and horizontal saccades. Exploratory saccade ratio shows the number of exploratory saccades divided by the total number of saccades per trial. Vertical to horizontal saccade ratio is calculated as the average y-components of a saccade divided by the average x-component. A ratio of 1 would indicate an equal amount of vertical and horizontal eye movements.

Appendix D - Statistical models and QQ-plots

This section lists full details on the models used for the statistical analysis of the real-world obstacle course results, as well as the QQ-plots used to visualize normal distribution of results.

Effect of Gaze Training (pre/post training condition) on trial duration

Model Specification:

- Model: Linear Mixed Model (lme)
- Dependent Variable: log(TrialDuration)
- Fixed Effects: PrePostTrainingCondition
- Random Effects: $\sim 1 +$ Participant | Participant
- Model Fit Statistics: AIC = -285.7289, BIC = -263.1566, logLik = 148.8645

Results:

- Intercept: Estimate = 3.567655, SE = 0.09670653, t-value = 36.89157, $p - value <$ 0.001
- PrePostTrainingCondition: Estimate = -0.177829, SE = 0.01577808, t-value = -11.27062, $p - value < 0.001$

Effects:

- Participant (Intercept): StdDev = 2.717010e-01
- Participant: StdDev (Intercept) correlation = 0
- Residual: StdDev = 1.411235e-01

Data Samples:

- Number of Observations: 320
- Number of Groups: 8

Effect of control phase (pre/post control condition) on trial duration

Model Specification:

- Model: Linear Mixed Model (lme)
- Dependent Variable: log(TrialDuration)
- Fixed Effects: PrePostControlCondition
- Random Effects: $\sim 1 +$ Participant | Participant
- Model Fit Statistics: AIC = -349.665, BIC = -327.0927, logLik = 180.8325

Results:

- Intercept: Estimate = 3.490358, SE = 0.10280603, t-value = 33.95090, $p - value <$ 0.001
- PrePostControlCondition: Estimate = -0.041963, SE = 0.01421692, t-value = -2.95161, $p - value = 0.0034$

Effects:

- Participant (Intercept): StdDev = 2.893858e-01
- Participant: StdDev (Intercept) correlation = 0
- Residual: StdDev = 1.271600e-01

Data Samples:

- Number of Observations: 320
- Number of Groups: 8

Effect of training phase (pre/post training condition) on number of collisions

Model Specification:

- Model: Negative Binomial Regression (glm.nb)
- Dependent Variable: Collisions
- Fixed Effects: PrePostTrainingCondition
- Random Effects: $\sim 1 +$ ParticipantID | ParticipantID

- Model Fit Statistics: Null Deviance: 291.34 on 319 degrees of freedom, Residual Deviance: 278.05 on 318 degrees of freedom, AIC: 762.19

Results:

- Intercept: Estimate = 0.02469, Std. Error = 0.12360, z value = 0.200, $p - value =$ 0.841657
- PrePostTrainingCondition: Estimate = -0.69315, Std. Error = 0.19145, z value = -3.621, $p - value = 0.000294$
- Random Effects: $\sim 1 + Participant \mid Participant$ (Not defined due to singularities)

Additional Information:

- Theta: Estimate = 0.681, Std. Error = 0.147
- Number of Fisher Scoring iterations: 1
- 2 x log-likelihood: -756.191

No estimates can be given for the random effect for this model. This is likely caused due to the number of subjects being too small.

Effect of control phase (pre/post control condition) on number of collisions

Model Specification:

- Model: Negative Binomial Regression (glm.nb)
- Dependent Variable: Collisions
- Fixed Effects: PrePostControlCondition
- Random Effects: $\sim 1 +$ Participant | Participant
- Model Fit Statistics: Null Deviance: 251.63 on 319 degrees of freedom, Residual Deviance: 251.37 on 318 degrees of freedom, AIC: 749.11

Results:

- Intercept: Estimate = -0.2469, Std. Error = 0.1500, z value = -1.645, $p - value =$ 0.0999
- PrePostControlCondition: Estimate = -0.1098, Std. Error = 0.2144, z value = -0.512, $p - value = 0.6085$
- Random Effects: $\sim 1 +$ ParticipantID | ParticipantID

Additional Information:

- Theta: Estimate = 0.4307, Std. Error = 0.0814
- Number of Fisher Scoring iterations: 1
- 2 x log-likelihood: -743.1110

No estimates can be given for the random effect for this model. This is likely caused due to the number of subjects being too small.

Effect of training phase (pre/post training condition) on head-centric DFoV

Model Specification:

- Model: Linear Mixed Model (lme)
- Dependent Variable: HeadCentricDFoV
- Fixed Effects: PrePostTrainingCondition
- Random Effects: $\sim 1 +$ Participant | Participant
- Model Fit Statistics: AIC = -345.4398, BIC = -324.6062, logLik = 178.7199

Results:

- Intercept: Estimate = 1.0161667, Std.Error = 0.01021678, DF = 231, t-value = 99.46056, $p - value < 0.001$
- PrePostTrainingCondition: Estimate = -0.0196667, Std.Error = 0.01444871, DF = 231, t-value = -1.36114, $p - value = 0.1748$

Effects:

- participant (Intercept): StdDev = 1.301521e-06
- participant: StdDev (Intercept) correlation = 0
- Residual: StdDev = 1.119192e-01

Data Samples:

- Number of Observations: 240
- Number of Groups: 8

Effect of control phase (pre/post training condition) on head-centric DFoV

Model Specification:

- Model: Linear Mixed Model (lme)
- Dependent Variable: HeadCentricDFoV
- Fixed Effects: PrePostControlCondition
- Random Effects: $\sim 1 +$ Participant | Participant
- Model Fit Statistics: AIC = -225.9001, BIC = -206.1403, logLik = 118.95

Results:

- Intercept: Estimate = 1.0163366, Std.Error = 0.01294036, DF = 193, t-value = 78.54007, $p - value < 0.001$
- PrePostControlCondition: Estimate = -0.0160366, Std.Error = 0.01834613, DF = 193, t-value = -0.87412, $p - value = 0.3831$

Effects:

- Participant (Intercept): StdDev = 1.442753e-06
- Participant: StdDev (Intercept) correlation = 0
- Residual: StdDev = 1.300490e-01

Data Samples:

- Number of Observations: 201
- Number of Groups: 7

Effect of training phase (pre/post training condition) on world-centric DFoV

Model Specification:

- Model: Linear Mixed Model (lme)
- Dependent Variable: WorldCentricDFoV
- Fixed Effects: PrePostTrainingCondition
- Random Effects: $\sim 1 +$ Participant | Participant
- Model Fit Statistics: AIC = -221.0613, BIC = -200.2277, logLik = 116.5307

Results:

- Intercept: Estimate = 0.97800, Std.Error = 0.01326768, DF = 231, t-value = 73.71298, $p - value < 0.001$
- PrePostTrainingCondition: Estimate = 0.06625, Std.Error = 0.01876333, DF = 231, t-value = 3.53082, $p - value = 0.0005$

Effects:

- Participant (Intercept): StdDev = $2.303544e - 06$
- Participant: StdDev (Intercept) correlation = 0
- Residual: StdDev = 0.1453401

Data Samples:

- Number of Observations: 240
- Number of Groups: 8

Effect of control phase (pre/post control condition) on world-centric DFoV

Model Specification:

- Model: Linear Mixed Model (lme)
- Dependent Variable: WorldCentricDFoV
- Fixed Effects: PrePostControlCondition
- Random Effects: $\sim 1 +$ Participant | Participant
- Model Fit Statistics: AIC = -90.34245, BIC = -70.58262, logLik = 51.17122

Results:

- Intercept: Estimate = 0.9865347, Std.Error = 0.01819139, DF = 193, t-value = 54.23087, $p - value < 0.001$
- PrePostControlCondition: Estimate = 0.0448653, Std.Error = 0.02579074, DF = 193, t-value = 1.73959, $p - value = 0.0835$

Effects:

- Participant (Intercept): StdDev = $4.234727e - 06$
- Participant: StdDev (Intercept) correlation = 0
- Residual: StdDev = 0.1828212

Data Samples:

- Number of Observations: 201
- Number of Groups: 7

QQ-plots

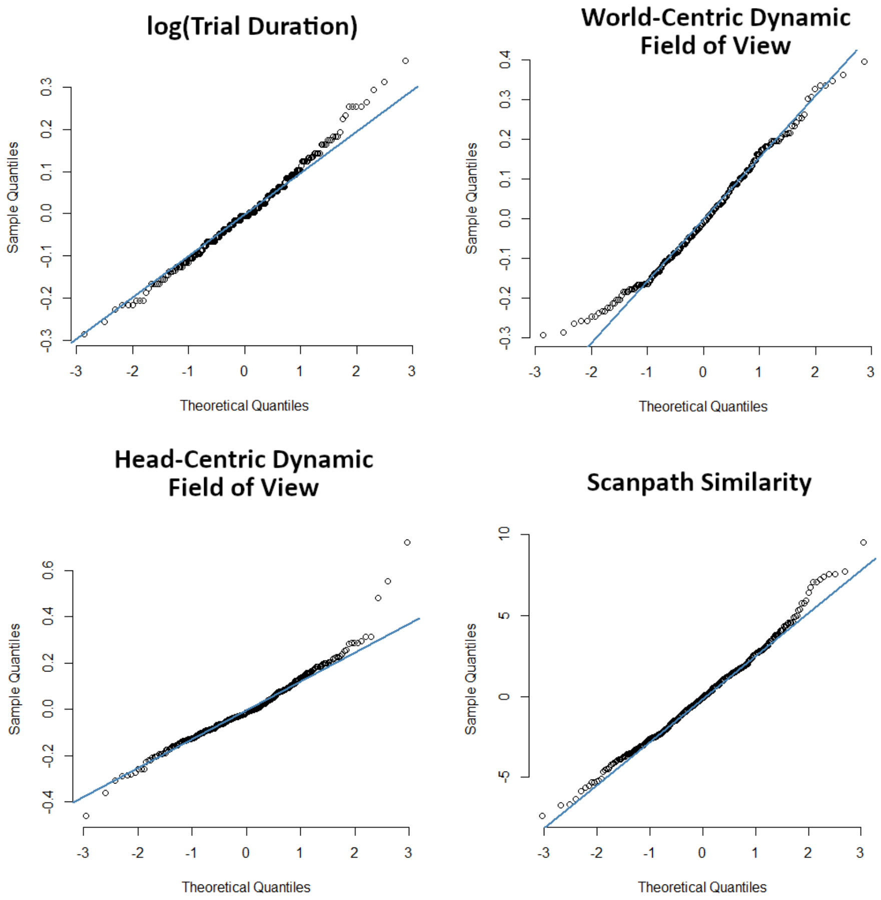

Figure 16: QQ-plots of the residuals of different result parameters of the real-world obstacle course.
